# Defining operational safety in clinical artificial intelligence systems

**DOI:** 10.1038/s41746-026-02450-7

**Published:** 2026-02-20

**Authors:** Young-Tak Kim, Hyunji Kim, Manisha Bahl, Michael H. Lev, Ramon Gilberto González, Michael S. Gee, Synho Do

**Affiliations:** 1 Department of Radiology, Massachusetts General Hospital, Harvard Medical School, Boston, MA USA; 2 Kempner Institute, Harvard University, Boston, MA USA; 3 KU-KIST Graduate School of Converging Science and Technology, Korea University, Seoul, Republic of Korea

**Keywords:** Cancer, Computational biology and bioinformatics, Mathematics and computing, Medical research

## Abstract

The clinical adoption of artificial intelligence (AI) has focused on enabling automation, but conventional accuracy metrics fail to answer a key question: when is it safe to trust an AI system? We introduce the Safety-Aware Receiver Operating Characteristic (SA-ROC) framework, which defines operational safety as an ability to meet pre-specified reliability levels. The SA-ROC curve delineates a Rule-in and a Rule-out Safe Zone, where autonomous action is permitted, and a Gray Zone, where human review is mandated. To quantify this non-automated workload, we introduce the Gray Zone Area (Γ_Area_), a metric measuring the operational cost of indecision. Our framework reveals a key reversal: in a case study of two FDA-cleared algorithms for cancer screening, the model with a statistically superior AUC was found to be operationally less safe for high-confidence screening. SA-ROC enables active governance, translating clinical policy into optimized workflows that inform operational safety and complement regulatory safety evaluation.

## Introduction

A paradox has emerged in clinical artificial intelligence (AI): despite hundreds of algorithms demonstrating high discriminative power in statistical benchmarks and achieving regulatory approval, their integration into routine care remains tentative^1–3^. The reason is not merely a lack of accuracy, but a significant gap between an AI’s validated statistical performance and the operational safety required for clinical trust, leaving a ‘validation debt’ that must be paid at the point of care^4,5^. This uncertainty presents clinicians with a significant challenge: navigating between reliance on an opaque algorithm and the risk of defensive over-testing, while remaining accountable for the AI’s potential errors^6–8^. This challenge in establishing trust is a key barrier to realizing the full potential of AI in safely automating cognitive tasks and reducing clinician burden^9^.

Contemporary methods offer valuable but partial guidance for safe automation: decision-curve analysis gauges clinical utility^10^, selective prediction enables abstention under cost trade-offs^11^, and conformal prediction provides finite-sample, distribution-free coverage of set-valued outputs^6^. Importantly, a growing body of work has begun to push conformal prediction beyond pure accuracy toward operational safety assurances, for example, delivering sample-efficient safety guarantees for robotic warning systems^12^, defining probabilistically certified safe regions in input space where local error rates are bounded^13^, and improving statistical reliability of interpretable rule-based classifiers via a novel score function for conformal prediction^14^. In medicine, conformal prediction has been applied to surface and manage uncertainty across tasks, flagging ambiguous cases or forming prediction sets for disease course monitoring^15^, reducing false alarms in clinical time-series^16^, and empirically validating reliability for medical image classification^17^. Nonetheless, these powerful methods often provide a set of heterogeneous analytical signals, such as coverage-valid label sets, spatially defined safe regions, abstention flags^18^, which leave clinicians to shoulder the cognitive burden of mentally integrating them into a concrete operating policy^6,7,19^. This lack of a unified operational directive presents a key translational gap between statistical validation and safe clinical automation^20,21^.

**Fig. 1:**
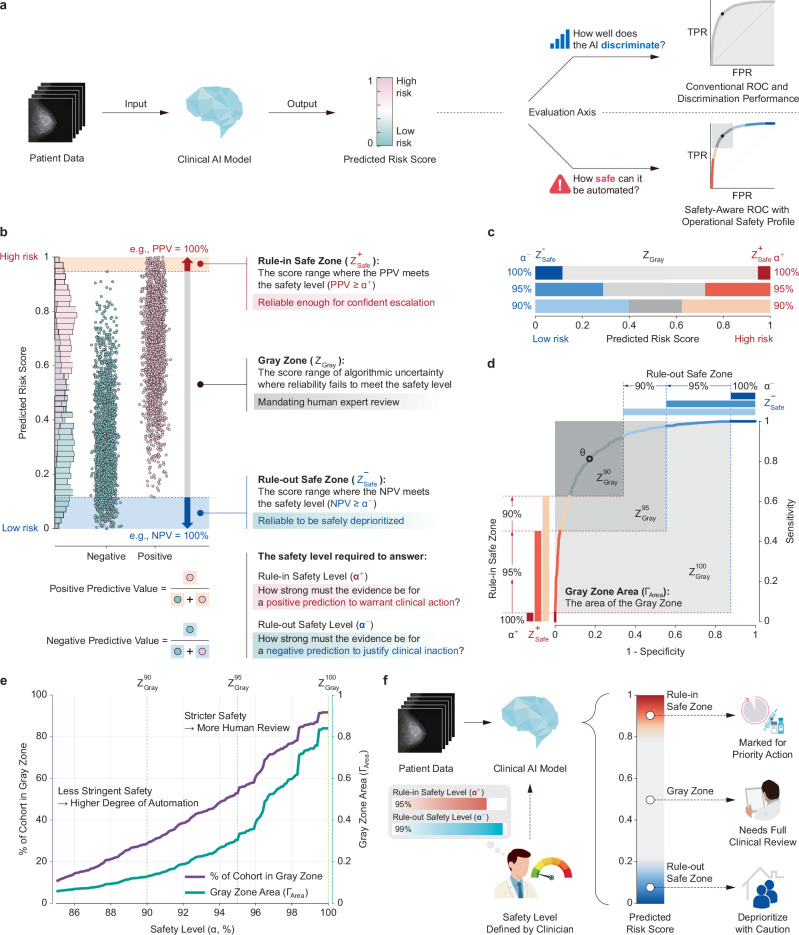
Conceptual diagram of the Safety-Aware ROC (SA-ROC) framework. **a** A standard AI model produces a continuous risk score (i.e., the predicted probability from a binary classifier) for each patient. This output can be evaluated along two distinct axes: its discriminative performance, often assessed using metrics like the area under the ROC curve, and its operational safety, which is visualized and quantified by the SA-ROC framework. **b** The framework partitions the model’s score distribution. Based on a user-defined safety criterion (*α*), it identifies a Rule-in Safe Zone (${Z}_{{\rm{Safe}}}^{+}$) at high scores and a Rule-out Safe Zone (${Z}_{{\rm{Safe}}}^{-}$) at low scores, and an intermediate Gray Zone (${Z}_{{\rm{Gray}}}$). **c** Score-range partitioning based on varying safety levels (*α*). Each bar demonstrates how increasing the required reliability shrinks the Safe Zones and expands the Gray Zone. **d** The SA-ROC curve visualizes the model’s operational landscape by color-coding segments according to their corresponding zones. The optimal threshold *θ* is indicated. The Gray Zone Area (Γ_Area_) is shown as a shaded rectangle in the top-left corner, providing an integrated view of both discrimination and operational uncertainty. **e** The Safety Profile Curve plots the Gray Zone proportion and Γ_Area_ across the safety levels (*α*), where *α* denotes *α*^+^ = *α*⁻ and asymmetric targets are written explicitly. This enables evaluation of the trade-off between safety and automation. **f** The framework maps AI outputs to a three-tiered clinical workflow: autonomous de-prioritization (Rule-out Safe Zone), essential human review (Gray Zone), and autonomous case escalation (Rule-in Safe Zone), thereby guiding clinical decisions and reducing cognitive burden.

To address this translational gap, we introduce the Safety-Aware Receiver Operating Characteristic (SA-ROC) framework, a tool designed specifically to synthesize these heterogeneous analytical signals into a unified operational directive. This synthesis enables safe, partial automation by setting clear, actionable trust conditions instead of imposing additional cognitive burden on clinicians (Fig. 1). In this work, we define operational safety as knowing the precise conditions under which a clinician can confidently act on an AI’s recommendation to rule in or rule out a disease. To translate this principle into a clear operational map, the framework uses a key hyperparameter: the safety level (*α*). The safety level (*α*) represents the clinical reliability target set by the clinician, indicating the minimum performance threshold required to establish actionable trust. It is defined by two specific objectives: the rule-in safety level (*α*^+^), which denotes the minimum acceptable positive predictive value (PPV) required to trust a rule-in action, and the rule-out safety level (*α*⁻), which denotes the minimum acceptable negative predictive value (NPV) required to trust a rule-out action.

The core function of the SA-ROC framework is to take these clinician-defined reliability targets (*α*^+^ and *α*^−^) as inputs, analyze the local data, and then automatically identify the specific prediction score thresholds required to achieve those targets. Thus, this process identifies two distinct score cutoffs: a high-score cutoff defining the start of the Rule-in Safe Zone (which satisfies *α*^+^) and a low-score cutoff defining the end of the Rule-out Safe Zone (which satisfies *α*⁻). Based on these data-driven thresholds, the framework then partitions the model’s predictions into three distinct zones. The first is the Rule-in Safe Zone, which represents a range of high-risk scores where the model’s performance meets or exceeds the specified reliability target (*α*^+^) for ruling in; cases in this zone are deemed reliable enough for autonomous escalation or action. The second is the Rule-out Safe Zone, which represents a range of low-risk scores where the model’s performance meets or exceeds the specified reliability target (*α*⁻) for ruling out; cases in this zone are deemed reliable enough for autonomous de-prioritization. The third is the Gray Zone, which corresponds to the intermediate range of uncertainty where the model’s reliability fails to meet either the rule-in (*α*^+^) or rule-out (*α*⁻) safety target, signifying that these cases are designated for mandatory human expert review. To quantify the operational burden of this non-automated cohort, the framework introduces the Gray Zone Area (Γ_Area_), a novel metric that allows stakeholders to evaluate the trade-off between the desired safety level (*α*) and the consequential cost of indecision.

This framework’s primary utility lies in its ability to facilitate a practical human-in-the-loop workflow, a process we term policy-driven automation. This workflow is not fixed; it is actively governed by the clinical policies derived within the framework. These policies can be established in two primary ways: either by directly choosing the safety level (*α*), or, for more complex scenarios, by defining a clinical utility function that balances the costs of errors against the benefits of correct decisions, allowing the framework to derive the optimal policy automatically. This process entails having the AI system autonomously handle all cases within the resulting Safe Zones (e.g., for autonomous de-prioritization or escalation), while the diagnostically challenging cases within the Gray Zone are, by design, escalated for human review. By translating clinical policy into AI behavior, the SA-ROC framework enables a critical shift from using AI as a static prediction tool to deploying it as a dynamically governed component of the clinical workflow.

In this paper, we demonstrate the framework’s effectiveness through extensive simulations and a real-world case study using two FDA-cleared AI solutions that are machine learning classifiers implemented as a convolutional neural network (CNN) and perform binary classification for cancer detection. By showing how diverse operational policies can be derived to meet specific clinical demands, this work offers a systematic framework for developing, governing, and validating clinical AI. To address the persistent gap between statistical performance and clinical trust, our ultimate goal is to provide the methodology required to establish operational trustworthiness for practical clinical automation.

## Results

### Score distribution profile determines operational safety

**Fig. 2:**
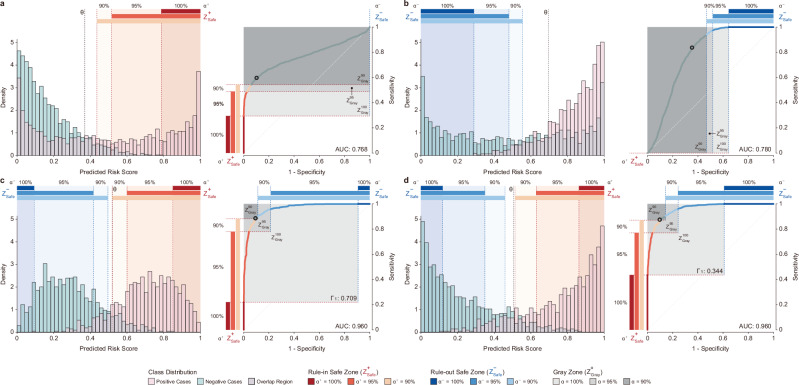
Simulated scenarios illustrating how score distribution shapes affect the Safety-Aware ROC (SA-ROC) curve. Each panel shows a model with a distinct operational safety profile, with specific AUC and the Gray Zone Area (Γ_Area_) values provided in the plots. Specifically, Γ_1_ denotes the Gray Zone Area at the maximal safety level (*α* = 100%). The plotted Gray Zones are computed under a symmetric setting, where *α* denotes *α*^+^ = *α*⁻, reflecting equal PPV and NPV targets. The circular marker on each ROC curve denotes the threshold *θ*, defined as the point maximizing the sum of sensitivity and specificity. **a** A model with a skewed distribution creating a strong Rule-in Safe Zone, suitable for confident case escalation. **b** A model with an opposing distribution skew, creating an expansive Rule-out Safe Zone ideal for safely excluding low-risk cases. **c** A high-AUC model with wide, overlapping distributions resulting in a large Gray Zone and high operational uncertainty. **d** A model with the same high AUC but narrow, well-separated distributions, resulting in a substantially smaller Gray Zone and more reliable automation.

A single metric like area under the curve (AUC) does not capture an AI’s full operational value, which is better understood through its distribution of prediction scores. To illustrate this, our simulation studies use the SA-ROC framework to analyze four distinct AI models, exhibiting how different score distribution profiles produce substantially different operational characteristics. For instance, a model with a modest AUC of 0.768 but with scores sharply peaked for positive cases established a robust Rule-in Safe Zone, making it effective for high-confidence confirmation tasks (Fig. 2a). Conversely, another model with a similar AUC of 0.780 but skewed towards negative cases produced an expansive Rule-out Safe Zone ideal for high-volume screening (Fig. 2b). Both scenarios demonstrate that significant operational value can emerge from models optimized for specific clinical functions, even when their overall performance is not top-tier. This principle becomes particularly important, however, when comparing models where aggregate metrics show no difference.

We contrasted two high-performing models with nearly identical AUCs of 0.960 and found their operational safety profiles diverged markedly under the most stringent conditions (*α* = 100%). The model with wider, overlapping distributions resulted in a large Gray Zone (Γ_Area_ = 0.709), leaving a high burden of non-automated workload (Fig. 2c). In stark contrast, the model with narrow, well-separated distributions yielded a substantially smaller Gray Zone (Γ_Area_ = 0.344), enabling a far greater degree of safe automation (Fig. 2d). These scenarios show that the SA-ROC framework can distinguish models with nearly identical AUCs but different capacities for safe automation. This positions the framework as a valuable diagnostic tool in the AI development lifecycle, providing the capability to dissect how a model’s intrinsic characteristics interact with a given data distribution, thereby identifying specific weaknesses and guiding targeted efforts in both model refinement and data curation.

### Performance reversal between FDA-cleared AI solutions

**Fig. 3:**
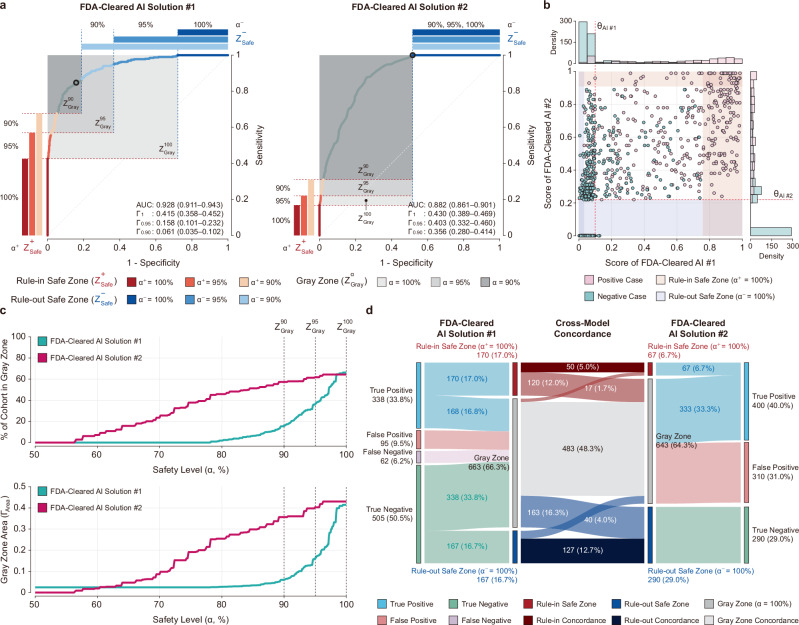
Comparative operational safety analysis of two FDA-cleared mammography AI solutions. **a** Safety-Aware ROC (SA-ROC) curves for AI Solution #1 and #2, Rule-in Safe (red), Rule-out Safe (blue), and Gray (gray) Zones defined at *α* = 90%, 95%, and 100%. AUC and Γ_Area_ are shown with 95% confidence intervals; specifically, Γ_1_, Γ_0.95_, and Γ_0.90_ denote the Gray Zone Areas at safety levels *α* = 100%, 95%, and 90%, respectively. Each ROC curve includes a circular marker indicating the predefined threshold *θ*, as provided by the respective AI vendors. **b** Scatter plot comparing predicted scores from both models, overlaid on a 2D Safe Zone grid at *α* = 100%, illustrating cross-model agreement and disagreement. Thresholds *θ*_AI #1_ and *θ*_AI #2_ are marked to indicate each model’s decision boundary. **c** Safety Profile Curves comparing the two models across levels ranging from 50% to 100%; top: percentage of cases in the Gray Zone; bottom: corresponding Γ_Area_ values. **d** Sankey diagrams showing case disposition at *α* = 100%, from reference standard (outside) to Safe Zone classification (center) for each model, with the center panel visualizing cross-model zone concordance. All zones and metrics are computed under a symmetric safety policy, where *α* denotes *α*^+^ = *α*⁻, indicating equal PPV and NPV thresholds.

A comparison of two FDA-cleared mammography AIs shows a notable performance reversal: the model with superior statistical performance was not the most operationally robust for high-confidence screening. Although AI Solution #1 demonstrated a higher AUC than AI Solution #2 (0.928 vs. 0.882; Fig. 3a, b), this advantage translated to better operational efficiency only for less stringent tasks, as evidenced by its more rapid Gray Zone reduction at moderate safety levels (*α* < 95%; Fig. 3c). However, this trade-off reverses when maximal safety is the priority. At the highest safety level of *α* = 100%, where no false negatives in the Rule-out Safe Zone are tolerated, the operational superiority of AI #2 emerges. AI #2 confidently and correctly ruled out 290 true negative cases, which safely removed 29.0% of the entire patient cohort from the radiologist’s workload. This represents a substantial improvement over AI #1, which cleared only 167 cases (16.7% of the cohort), with a difference of 123 cases in the Rule-out Safe Zone in favor of AI #2 (95% confidence interval: 87.0–150.0). Crucially, 163 of the cases that AI #1 left in its uncertain Gray Zone were confidently ruled out by AI #2 (Fig. 3d). This result shows that the AUC can be misleading and that models should be evaluated for their operational safety to understand their true clinical utility. Furthermore, this granular analysis suggests the potential for synergistic implementation strategies. For instance, a hybrid workflow could first leverage AI #2 for its superior high-confidence rule-out capability to reduce the initial workload, and then triage the remaining, more ambiguous cases with AI #1, potentially capitalizing on the distinct strengths of both models. Additionally, to assess generalizability, we analyzed operational stability across varying prevalence conditions (1%–40%) to characterize the distinct stability profiles of each model and to investigate the dynamics of the performance reversal in the Rule-out Safe Zone (*α*⁻ = 100%) (see Supplementary Note 1).

### Patterns of human diagnostic behavior in AI Safe Zones

**Fig. 4:**
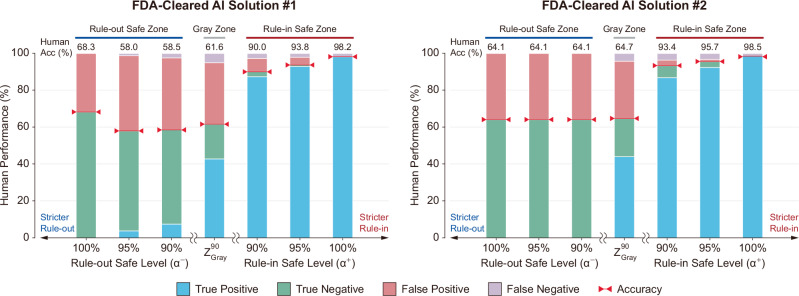
Analysis of human radiologist performance within safety and Gray Zones of the FDA-cleared AI solutions. The charts show the proportional diagnostic outcomes of human assessments on cases stratified by the Safe Zones of AI Solution #1 and #2. For each model, cases are grouped into Rule-in Safe Zones (*α*^+^ = 100%, 95%, 90%), the Gray Zone defined under the *α* = 90% safety level (${Z}_{{\rm{Gray}}}^{90}$), and Rule-out Safe Zones (*α*⁻ = 100%, 95%, 90%). The stacked bars are colored by the radiologist’s outcome: true positive (blue), true negative (green), false positive (red), and false negative (purple). Red triangles and corresponding numeric labels indicate the overall human accuracy within each zone.

By stratifying cases into distinct zones of reliability, the SA-ROC framework provides a new perspective to understand the decision-making patterns of human radiologists and compare them with the AI’s safety assessment. Our analysis revealed the most critical human-AI discordance in the Rule-out Safe Zone (Fig. 4). Here, where the AI was most confident that cases were low-risk (e.g., *α*⁻ = 100%), human accuracy was paradoxically at its lowest. This was driven by a high rate of false positives. In other words, radiologists frequently flagged these AI-identified safe cases as suspicious, a tendency often described as diagnostic over-calling in high-stakes screening. The framework identifies a large cohort where the AI’s reliable assessment can help counterbalance a clinical bias towards over-investigation, potentially reducing unnecessary recalls.

Conversely, the other zones validated the framework’s logic through strong human-AI alignment. In the Rule-in Safe Zone, the high accuracy of human radiologists (e.g., above 98% at *α*^+^=100%) confirmed that the AI successfully identified cases with a high likelihood of disease. The Gray Zone functioned as a domain of shared uncertainty where both AI and human experts struggled, affirming its role as the designated area requiring essential, focused human review. This stratification provides a practical blueprint for calibrating clinical trust by showing where AI can safely de-escalate care and where human expertise remains essential.

### Operational safety profiles for policy-driven automation

**Fig. 5:**
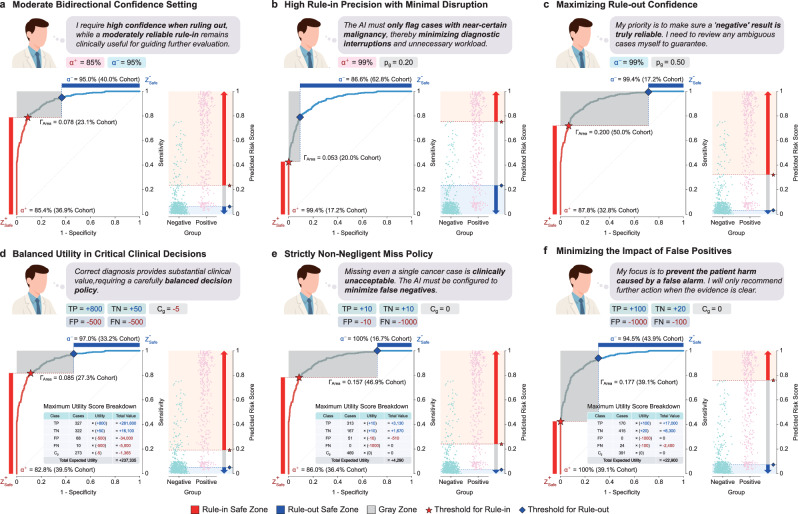
Operational safety profiles of AI Solution #1 under varying clinician-defined policies. **a–c** Policies defined by explicit constraints on reliability. Each panel shows how the same AI model behaves when its Safe Zones are customized to different clinician-defined safety levels (*α*^+^ and *α*⁻). **d–f** Policies derived from utility maximization. These panels show the optimal policy and corresponding thresholds derived by maximizing a pre-defined utility function. The table within each panel details this optimal policy, listing the outcome class, the number of cases for that outcome, the pre-defined per-case utility value, the calculated total value (Cases × Utility), and the final total expected utility (the sum of all total values). Within all panels, the ROC curve is color-coded to indicate the Rule-in Safe Zone (red), Rule-out Safe Zone (blue), and Gray Zone (gray). The thresholds for rule-in and rule-out are marked by a star and a diamond, respectively. The specified safety levels (*α*^+^ and *α*⁻) are labeled, with the percentage of the cohort in each corresponding zone shown in parentheses. The shaded rectangle in the top-left corner represents the Gray Zone Area (Γ_Area_). TP: true positive, TN: true negative, FP: false positive, FN: false negative, *C*_g_: cases deferred to the Gray Zone, *p*_g_: proportion of the cohort deferred to the Gray Zone.

A key contribution of the SA-ROC framework is its ability to translate explicit institutional rules and priorities into quantifiable AI behavior, enabling the AI to function as a dynamically governed component of the clinical workflow. The framework first demonstrates this capability by forging policies from direct clinical constraints. While a balanced policy serves as a baseline by meeting moderate bidirectional confidence targets (Fig. 5a), the framework’s flexibility becomes particularly evident when accommodating specialized, competing demands. For instance, a policy tailored for high-precision specialist referrals (Fig. 5b) enforces a stringent PPV of over 99%, which directly trades off against rule-out confidence. Conversely, a policy designed for high-volume screening (Fig. 5c) mandates near-perfect rule-out confidence (NPV ≥ 99%), thus codifying a different clinical priority at the expense of PPV.

Beyond direct rules, the framework navigates more complex scenarios by maximizing a clinical utility function that weighs the nuanced costs of errors against the benefits of correct decisions. While a balanced utility function (Fig. 5d) offers a sensible default optimization, the framework’s utility is particularly evident in its ability to resolve policies with stringent clinical mandates. Faced with a ‘strictly non-negligent’ policy where missing a cancer is unacceptable (Fig. 5e), the optimal strategy becomes highly conservative: to achieve the target of 100% NPV, the AI eliminates its ability to rule out any case, deferring all but the most certain positive calls to human review. In stark contrast, a policy engineered to minimize the harm from false positives (Fig. 5f) compels the AI to be exceptionally cautious, achieving the target of 100% PPV by sacrificing broad detection for absolute certainty. These scenarios show the SA-ROC framework functioning as a versatile governance tool, enabling stakeholders to translate specific clinical, economic, or ethical priorities into predictable and transparent AI policies.

## Discussion

A fundamental question from clinicians grappling with AI is not simply “How accurate is it?” but rather, “Under which conditions, and to what extent, can I safely trust its judgment?” Our case study comparing two FDA-cleared mammography AI solutions helps address this question by providing a quantitative method for assessing operational safety. The higher-AUC model was the less robust choice for high-volume screening requiring near-perfect reliability. This insight, often overlooked by conventional metrics, reveals a critical gap between statistical validation and clinical safety that impedes AI adoption^4,22,23^. The SA-ROC framework was designed to bridge this gap and provide a basis for actionable trust. It moves beyond simply evaluating a model’s operational safety to guiding its safe deployment, enabling users to define the precise conditions under which an AI can be trusted to meet specific clinical demands. This may help shift the conversation around AI from a focus on statistical superiority toward a practical dialogue on operational safety and trustworthiness.

The SA-ROC framework augments the traditional receiver operating characteristic (ROC) curve^24^, reframing it from a static plot of statistical performance into a dynamic, visual map of a model’s operational safety landscape. This approach is situated within a broader field of advanced ROC methodologies. For example, some methods emphasize top-ranked predictions (concentrated ROC)^25^ or weight the clinical importance of findings (weighted alternative free-response ROC)^26^. However, the primary objective of SA-ROC is fundamentally different. These established methods typically aim to refine the evaluation of a model’s intrinsic properties. In contrast, the SA-ROC framework’s central innovation is not to better assess a model’s inherent performance, but to link its output directly to an extrinsic, clinician-defined safety policy. Instead of analyzing a fixed aspect of the curve, our framework allows users to impose a policy-driven mandate, such as “a negative finding must be at least 99% reliable”, which then actively partitions the entire ROC space. This partitioning creates three distinct operational zones: a Rule-in Safe Zone and a Rule-out Safe Zone, where the AI can be trusted for autonomous action, and the intermediate Gray Zone, which designates where human expertise remains indispensable.

The evaluation of medical AI is shifting from a focus on predictive accuracy toward trustworthiness and the communication of uncertainty^27^. The SA-ROC framework contributes to this evolving field by serving as an important translational bridge between established statistical theories of uncertainty and the practical demands of operational safety. Indeed, the ability for a model to say, “I don’t know” and abstain from prediction when uncertain is a necessary capability for safe clinical deployment^6^. This has spurred the development of new safety paradigms such as zero-error tolerance, which aims to set new standards for reliability by having AI operate only in scenarios where errors can be minimized^28^. This trend extends even to foundational tasks like data annotation, where systems can automatically generate reliable labels based on quantitative similarity to an internal reference atlas^29^. In parallel, for clinical deployment, various frameworks have been developed to enable a model to abstain from prediction. For example, algorithmic uncertainty has been incorporated into deep learning models to provide ‘no prediction’ options for highly uncertain clinical cases^30^. Several statistical frameworks also have been developed, such as selective prediction, which provides rules for a model to abstain based on the potential cost of an error^11,31^, while conformal prediction generates a set of possible outcomes with a rigorous guarantee that the set will contain the true answer in a pre-specified percentage of the cases (e.g., 95%)^12,14,32^. Building on the principles of conformal prediction, probabilistic safety regions extend such probabilistic guarantees to the input space, providing bounded error regions with defined confidence levels^13^. However, while these methods provide a robust statistical method for a model to abstain, a gap remains in translating their outputs into an actionable clinical policy. The SA-ROC framework is designed to bridge this exact gap. To further clarify the conceptual distinctions, Table 1 summarizes the comparative characteristics of SA-ROC, selective prediction, conformal prediction, and probabilistic safety regions in terms of their objectives, key parameters, and decision-level outputs. While selective prediction typically optimizes the risk-coverage trade-off^31^ or minimizes global costs^11^ via unidirectional rejection, SA-ROC enforces bidirectional safety constraints. It treats clinical reliability targets (e.g., PPV ≥ 95%) not as variables to be traded off for efficiency, but as non-negotiable constraints that simultaneously define Safe Zones for both Rule-in and Rule-out. Similarly, while conformal prediction provides rigorous statistical coverage through dynamic prediction sets^33^ (e.g., variable set sizes per patient), SA-ROC operationalizes these signals into static triage zones. By converting variable outputs into definitive actions (e.g., “Automated Rule-out” vs. “Manual Review”), our framework enables the establishment of standardized clinical protocols. This operational logic is anchored by the primary hyperparameter, *α*, which serves not as a mere statistical significance level, but as the explicit threshold for these safety policies. This reframes the problem: the Gray Zone is no longer just a region of statistical ambiguity but the direct result of an explicit safety policy. Our framework, therefore, does not replace these statistical methods but connects their principles to clinical reality, making their abstract guarantees operationally meaningful. This design effectively decouples the clinical safety policy from the underlying statistical mechanics, establishing SA-ROC as a modular governance layer capable of evolving with advanced estimators. For instance, future implementations could utilize conformal risk control algorithms^34^ to derive zone thresholds, thereby combining SA-ROC’s operational utility with rigorous finite-sample guarantees for intrinsic error rates.

**Table 1:** Comparison of SA-ROC with related uncertainty-aware prediction frameworks

Aspect	SA-ROC Framework	Selective Prediction	Conformal Prediction	Probabilistic Safety Regions
Primary goal	Operational safety under a clinician-defined policy	Risk–coverage optimization under a target coverage or abstention cost	Marginal coverage guarantee for a set of possible labels	Probabilistic certification of a low-error region in input space
Key hyperparameter	Clinical reliability target (e.g., 95% PPV, 99% NPV)	Target coverage or abstention Cost (e.g., cover 80% of cases)	Significance level (e.g., 5% error rate)	Error probability bound with confidence (e.g., 1% error with 95% confidence)
Output for clinical decision	Three-zone workflow (Rule-in, Rule-out, Gray Zone)	Binary decision for each case: Predict or Abstain	A set of possible labels for a given case	Certified subset of cases where model predictions are trusted
Core question addressed	Under what conditions is it safe to trust the AI for a clinical task?	Given the risk/cost, should the AI make a prediction for this case?	Which set of labels is guaranteed to contain the truth for this case?	Which types of cases can be safely predicted with guaranteed low error?
Key distinction of SA-ROC	Decouples safety policy from architecture to enforce bidirectional constraints on any scoring model.	Enforces safety as a non-negotiable constraint regardless of workload, avoiding SP’s safety-efficiency trade-off.	Provides static operational zones for definitive action, avoiding CP’s interpretation-heavy dynamic sets.	Operates as a governance layer on output scores, unlike PSR’s dependency on specific adjustable classifiers.

*CP* conformal prediction, *NPV* negative predictive value, *PPV* positive predictive value, *PSR* probabilistic safety regions, *SP* selective prediction.

Beyond visualization, SA-ROC quantifies deferred workload via Γ_Area_, enabling workload and automation planning. This cost has significant implications for all stakeholders: for clinicians, it predicts the daily workload from deferred cases; for health systems, it represents the economic toll of logistical bottlenecks; and for developers, it provides a concrete target for shrinking a model’s operational uncertainty. Clinically, the magnitude of Γ_Area_ serves as a direct indicator of the operational cost required to maintain the desired safety level. A small Γ_Area_ suggests that the model can autonomously handle the majority of cases with high confidence, leading to a streamlined workflow and high automation efficiency. Conversely, a large Γ_Area_ indicates a significant proportion of ambiguous cases where the model cannot guarantee safety. In such scenarios, the framework signals a need for increased human resource allocation, as these gray cases must be diverted to expert clinicians for manual review to prevent medical errors. This cost is not merely theoretical; recent empirical studies have demonstrated its tangible impact on physicians. For example, AI tools lacking transparent uncertainty management have been shown to increase radiologists’ burnout risk^35^ and paradoxically increase the time physicians spend reviewing information rather than reducing it^36^. Strategically managing this cost is therefore crucial, and the framework enables this by revealing the inherent trade-off between the desired safety level (*α*) and the size of the Gray Zone. The Safety Profile Curve (Fig. 1e) illustrates the trade-off between *α* and Gray Zone size, aiding in the selection of an elbow point that maximizes reliability without disproportionately increasing workload. Models with small Γ_Area_ at high *α* levels are better suited for safe automation in clinical workflows. Consequently, this single metric enables a transparent dialogue about the trade-offs between safety and automation efficiency.

The framework extends beyond simply quantifying uncertainty to actively govern an AI’s behavior, offering a path for it to evolve from a static tool toward an adaptable component in clinical practice. Our framework makes this governance possible by enabling the formulation of optimal policies tailored to specific clinical contexts, achieved through either explicit constraints or utility maximization. This policy-driven capability is crucial because it resists the one-size-fits-all fallacy that often hinders AI adoption, where a single model configuration is expected to perform optimally across diverse clinical settings^37,38^. Our prevalence simulation analysis (see Supplementary Note 1) provides a clear example, demonstrating how two high-performing AI solutions respond in vastly different ways to shifts in epidemiological context. We found that a model’s operational stability is not a fixed property but is critically linked to its intrinsic score distribution, reinforcing the need for local, policy-driven validation. By enabling institutions to deploy the same validated AI model under distinct, locally-optimized policies, our framework can help shift AI integration from a purely technical challenge toward a transparent process of clinical governance, where operational reality is explicitly shaped by declared values.

Our work has several limitations that open new avenues for research. First, a primary limitation of our study pertains to its validation scope. The generalizability of the SA-ROC framework represents an important area for future research. This validation is particularly important because the Safe Zones are defined by prevalence-dependent metrics (PPV and NPV). We performed an initial assessment of this effect using a bootstrap-based simulation on our dataset to analyze operational stability under varying prevalence conditions. However, this simulation does not replace the need for real-world validation. Even with resampling, our analysis remains constrained by the retrospective dataset of 1000 cases. At high *α* levels (e.g., nearing 100%), thresholds rely on the sparse tails of the prediction score distribution, leading to potential instability that bootstrap intervals may not fully mitigate. Therefore, evaluation on large-scale, multi-center datasets is essential for calibrating the framework and defining its optimal role in real-world clinical workflows. Second, prospective validation through user studies is essential to understand clinical interaction, workflow impact, cognitive load, and potential biases such as over-reliance on the Safe Zone designation. Third, while the user-defined safety level (*α*) allows clinical context specificity, its variability complicates standardized model comparisons and regulatory agreement, highlighting the need for establishing benchmark *α* values via expert consensus. Fourth, the current Γ_Area_ metric captures total operational uncertainty, encompassing both data and model sources. Future work could aim to disentangle these factors through controlled comparative analyses to determine whether the operational burden stems primarily from input data complexity or model limitations. Finally, while our study focuses on binary classification, future research will adapt the SA-ROC framework to complex tasks such as multi-class problems and large language model assessments. Such efforts could contribute to the development of robust operational safety standards for a wider range of clinical AI. A long-term goal of this research is to contribute to the development of widely adopted standards for translating statistical performance into demonstrable operational safety for clinical AI applications.

Looking ahead, SA-ROC is designed to do both: automate what is safe and learn from what is uncertain. This dual capability allows it to advance clinical practice while also facilitating AI model refinement and scientific discovery. The impact of our framework could be realized through its potential to reshape clinical workflows. By identifying a large volume of normal cases, the framework enables significant changes to clinical practice and the nature of the human-AI partnership. For instance, in high-resource settings, it enables a safe, partial automation workflow: the AI autonomously handles the vast majority of cases in the Safe Zones, allowing specialists to focus their expertise only on complex cases deferred to the Gray Zone, thereby enhancing throughput and mitigating burnout. This impact could be significant in low- and middle-income countries, where a validated safe AI can empower trained healthcare workers to manage most negative cases, democratizing access to high-quality diagnostics^39–41^. Explicitly defined Safe Zones also have the potential to reshape the human-AI interface by filtering out low-risk safe cases, potentially mitigating alert fatigue^42^ and potentially reducing clinical decision fatigue^43^. However, this new dynamic introduces important challenges, such as automation bias^44^ and legal liability^45^ if rare errors occur. To mitigate this specific risk of over-reliance within ‘Safe Zones’, actual clinical deployment must prioritize human-AI interaction designs that go beyond passive display. Specifically, interfaces can incorporate cognitive forcing functions^46^ such as ‘on-demand’ access where the safety status remains hidden until explicitly requested, ‘update’ protocols requiring clinicians to input a provisional diagnosis before viewing the AI output, or ‘wait’ mechanisms that introduce a mandatory delay. These interventions are designed to disrupt heuristic reliance and compel analytical thinking before the AI’s safety status is accepted. Conversely, labeling a case as part of the Gray Zone may heighten clinician vigilance but could introduce cognitive burdens if not thoughtfully implemented^47,48^, emphasizing that successful integration of safe AI is ultimately a human-centered design challenge where interface and workflow are as critical as the algorithm itself. Therefore, future prospective studies are essential to understand these deeper cognitive and psychological impacts.

In parallel, our framework reframes the Gray Zone from a model failure into a resource for model improvement and scientific discovery. By definition, the cases that consistently fall into this zone are the most ambiguous and diagnostically challenging, representing the limits of the current model’s knowledge. Systematic analysis of this cohort offers a powerful opportunity for a continuous learning loop^49,50^. For developers, it pinpoints a model’s specific weaknesses, enabling targeted data augmentation and feature engineering to ‘shrink’ the clinically-relevant uncertainty. Employing the Gray Zone as a targeted dataset enables more sophisticated model improvement strategies, such as directly integrating its size into the loss function for minimization or optimizing the model’s policy by incorporating the utility values for each outcome as a reward signal within a reinforcement learning framework. Furthermore, for researchers, this cohort may highlight atypical disease presentations or rare subtypes that represent gaps in current clinical understanding itself, thus pointing toward new avenues of medical research. In this light, the Gray Zone can be reframed from a cost of indecision into a resource for innovation, creating a positive feedback loop for improving both AI models and clinical knowledge.

## Methods

### The Safety-Aware ROC framework

**Fig. 6:**
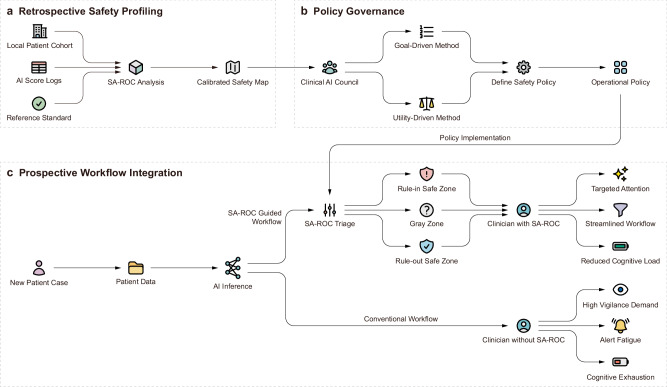
The SA-ROC end-to-end operational framework: from retrospective calibration to prospective workflow integration. This diagram illustrates the three stages of the framework: **a** Retrospective safety profiling. **b** Policy governance. **c** Prospective workflow integration. The framework first generates a calibrated safety map from local data, which allows clinical decision-makers to define an operational policy. This policy is then implemented to augment each new AI inference, enabling a safer, more targeted human-AI interaction and reducing cognitive load compared to conventional workflows.

The SA-ROC framework is a visual-analytic tool designed to augment the traditional ROC curve with operational safety information. It provides a structured approach to move beyond evaluating a model’s statistical performance by visualizing its operational safety in a clinical context. We conceptualize this as a complete end-to-end operational framework (Fig. 6), which consists of three key stages. First, a retrospective safety profiling stage uses local data to analyze a model and generate a calibrated safety map, which represents the comprehensive set of Zone area information corresponding to *α*^+^ and *α*⁻ across all possible risk scores and thresholds (Fig. 6a). Second, a policy governance stage allows clinical decision-makers to use this map to define a formal, binding operational policy (Fig. 6b). Third, a prospective workflow integration stage implements this policy to triage new cases, contrasting with conventional workflows to streamline human-AI interaction and mitigate cognitive load (Fig. 6c). The framework achieves this by systematically partitioning the model’s range of prediction scores into distinct zones based on clinician-defined reliability criteria. This process also enables the active design of AI decision policies tailored to specific clinical needs.

### Defining the Safe Zones and the Gray Zone

The framework’s safety assessment is built upon two clinically intuitive metrics: PPV, the probability that a positive prediction is correct, and NPV, the probability that a negative prediction is correct. To translate these metrics into a formal policy, the framework uses clinician-defined safety levels. The framework defines two distinct safety targets. The rule-in safety level (*α*^+^) is the minimum acceptable PPV required for a positive prediction. The rule-out safety level (*α*⁻) is the minimum acceptable NPV for a negative prediction.

A clinical safety policy is established by setting specific values for these levels (e.g., *α*^+^ ≥ 95% and *α*⁻ ≥ 99%). Based on these clinician-defined targets, the framework partitions the model’s predictions into three operational zones:

- The Rule-in Safe Zone (${Z}_{{\rm{Safe}}}^{+}$) consists of high-risk scores where the model’s PPV meets or exceeds the specified *α*^+^ target, indicating these cases are reliable enough for autonomous escalation or clinical action under the defined safety policy.
- The Rule-out Safe Zone (${Z}_{{\rm{Safe}}}^{-}$) consists of low-risk scores where the model’s NPV meets or exceeds the specified *α*⁻ target, permitting automated deprioritization under the defined safety policy.
- The Gray Zone (${Z}_{{\rm{Gray}}}$) is the intermediate range of scores where the model’s reliability fails to meet either the rule-out (*α*⁻) or rule-in (*α*^+^) safety level. These predictions are considered uncertain and require essential human expert review.

The formal definitions of the Safe Zones are established using the following notation. Let *S* be the complete set of samples in a dataset. For each sample *i* ∈ *S*, the binary classification model assigns a continuous risk score *s*_*i*_ ∈ [0, 1], representing the model’s predicted probability. Each sample has a corresponding true class label *y*_*i*_, where *y*_*i*_ ∈ {*C*_1_, *C*_0_} for the positive and negative classes, respectively. The zones are defined by identifying the set of scores, *s*, that satisfy the safety criteria by searching over all possible decision thresholds, *τ*′. Specifically, we performed an empirical linear scan over all unique, sorted prediction scores in the dataset to identify the precise boundaries without discretization gaps. This search aims to find the boundaries that satisfy the clinician-defined rule-in safety level (*α*^+^) and rule-out safety level (*α*⁻). The search for these boundaries utilizes the infimum (inf) and supremum (sup) operators to find the greatest lower bound and least upper bound of the set of qualifying thresholds. By utilizing these operators, the framework enforces a monotonicity constraint, ensuring that the resulting Safe Zones are defined as contiguous intervals bounded by a single threshold. Logical conditions are evaluated using the indicator function, *I*(⋅), which returns 1 if a condition is true and 0 otherwise.

Based on this notation, the Safe Zones are formally defined as:

Rule-in Safe Zone (${Z}_{{\rm{Safe}}}^{+}$): The set of scores s where the PPV is at least the rule-in safety level, *α*^+^.

1 $${Z}_{{\rm{Safe}}}^{+}=\left\{s \mid s\ge \inf \left\{{\tau }^{{\prime} }\mid \frac{{\sum }_{i\in S}I\left({s}_{i}\ge {\tau }^{{\prime} }\,{\rm{and}}\,{y}_{i}={C}_{1}\right)}{{\sum }_{i\in S}I\left({s}_{i}\ge {\tau }^{{\prime} }\right)}\ge {\alpha }^{+}\right\}\right\}$$

Rule-out Safe Zone **(**${Z}_{{\rm{Safe}}}^{-}$**)**: The set of scores s where the NPV is at least the rule-out safety level, *α*⁻.

2 $${Z}_{\mathrm{Safe}}^{-}=\left\{s \mid s< \sup \left\{{\tau }^{{\prime} }\mid \frac{{\sum }_{i\in S}I\left({s}_{i}<{\tau }^{{\prime} }\,\mathrm{and}\,{y}_{i}={C}_{0}\right)}{{\sum }_{i\in S}I\left({s}_{i}<{\tau }^{{\prime} }\right)}\ge {\alpha }^{-}\right\}\right\}$$

Gray Zone **(**${Z}_{{\rm{Gray}}}$**)**: The set of scores s not included in either Safe Zone.

3 $${Z}_{\mathrm{Gray}}=\{s\mid s\,\notin \,{Z}_{\mathrm{Safe}}^{+}\cup {Z}_{\mathrm{Safe}}^{-}\}$$

### Quantifying the non-automated workload with the Gray Zone

To provide a single, quantitative measure of a model’s operational uncertainty, we introduce the Gray Zone Area (Γ_Area_). This index directly measures the size of the Gray Zone in the ROC space, representing the clinical workload or ‘cost of indecision’ that the AI model imposes. Alongside this, the percentage of the total cohort that falls into the Gray Zone serves as another intuitive metric to directly quantify the non-automated workload. A model with a smaller Γ_Area_ is more operationally efficient because it safely automates a larger portion of the workload while meeting the specified safety criteria. The framework also utilizes a Safety Profile Curve, which plots both the percentage of the cohort in the Gray Zone and the Γ_Area_ against a range of safety levels (*α*). This curve visualizes the trade-off between safety and workload, allowing stakeholders to select an optimal operational policy.

**Fig. 7:**
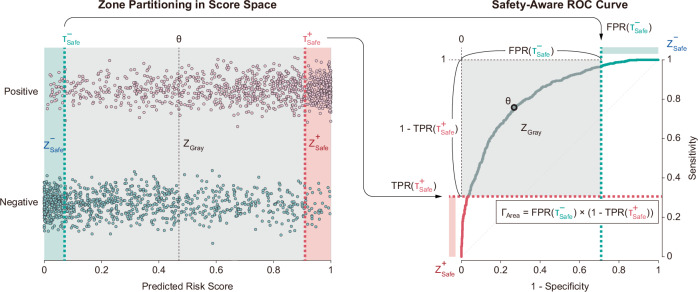
Illustrative computation of Γ_Area_ mapping from score space to ROC space. The scatter plot visualizes the distribution of predicted risk scores for positive (pink circles) and negative (green circles) cases, where the score range is partitioned into three operational zones—Rule-out Safe (${Z}_{{\rm{Safe}}}^{-}$), Gray ${(Z}_{{\rm{Gray}}}$), and Rule-in Safe (${Z}_{{\rm{Safe}}}^{+}$)—defined by the safety thresholds (${\tau }_{{\rm{Safe}}}^{-}$ and ${\tau }_{{\rm{Safe}}}^{+}$). Arrows illustrate the direct mapping of these thresholds to the ROC space, where the rule-out threshold (${\tau }_{{\rm{Safe}}}^{-}$) defines the boundary for the false positive rate (FPR(${\tau }_{{\rm{Safe}}}^{+}$)) and the rule-in threshold (${\tau }_{{\rm{Safe}}}^{+}$*)* defines the boundary for the true positive rate (TPR(${\tau }_{{\rm{Safe}}}^{+}$)). The Gray Zone Area (Γ_Area_) is computed as the geometric area of the shaded rectangle formed by these coordinates in ROC space. The standard optimal threshold is denoted by *θ*.

The Gray Zone Area (Γ_Area_) quantifies the size of the uncertain region in the ROC space (Fig. 7). Let ${\tau }_{{\rm{Safe}}}^{+}$ be the infimum (lowest score) of the Rule-in Safe Zone and ${\tau }_{{\rm{Safe}}}^{-}$ be the supremum (highest score) of the Rule-out Safe Zone. The Γ_Area_ is the area of the rectangle in ROC space defined by the false positive rate (FPR) at the rule-out boundary (${\tau }_{{\rm{Safe}}}^{-}$) and the true positive rate (TPR) at the rule-in boundary (${\tau }_{{\rm{Safe}}}^{+}$):

4 $${\Gamma }_{\mathrm{Area}}=\mathrm{FPR}\left({\tau }_{\mathrm{Safe}}^{-}\right)\times \left(1-\mathrm{TPR}\left({\tau }_{\mathrm{Safe}}^{+}\right)\right)$$

### Visualization of the safety landscape on the ROC curve

The core of our method is the visualization of this safety landscape on the ROC curve. The SA-ROC curve is constructed by color-coding the segments of the standard ROC curve based on the zone to which their corresponding threshold (*τ*), the cutoff applied to the model’s continuous risk scores to make binary predictions, belongs. Segments where the threshold *τ* falls within the Safe Zones (${Z}_{{\rm{Safe}}}^{+}$ or ${Z}_{{\rm{Safe}}}^{-}$) are colored to indicate safety, with different colors potentially representing different safety levels (e.g., *α* = 90%, 95%, 100%). For these visualizations, we employ a standard SA-ROC configuration. In this setting, we use a symmetric safety parameter *α* (implying *α*^+^ = *α*⁻) to standardize the evaluation of operational uncertainty across uniform tiers, whereas the framework fundamentally treats these parameters as operationally independent values to be tuned according to specific clinical needs. Segments where the threshold *τ* falls within the Gray Zone (${Z}_{{\rm{Gray}}}$) are colored with a neutral color (e.g., gray) to visually represent uncertainty. This visualization provides an intuitive and immediate understanding of a model’s operational characteristics, showing not only how well it discriminates but also where on its decision spectrum it is safe to use.

To explicitly quantify the operational cost of safety, the framework further generates the Safety Profile Curve. This curve plots the magnitude of the Gray Zone as a function of the target safety level, thereby visualizing the trade-off between reliability stringency and clinician workload. This curve is also generated using the standard SA-ROC configuration (*α*^+^ = *α*⁻), providing a streamlined view of how the operational cost scales with a unified safety target.

### Deriving operational policies from clinical goals

A key capability of the SA-ROC framework is its ability to actively shape an AI’s operational policy rather than just passively evaluate it. This is achieved through two primary paradigms for translating clinical priorities into a formal policy.

**Fig. 8:**
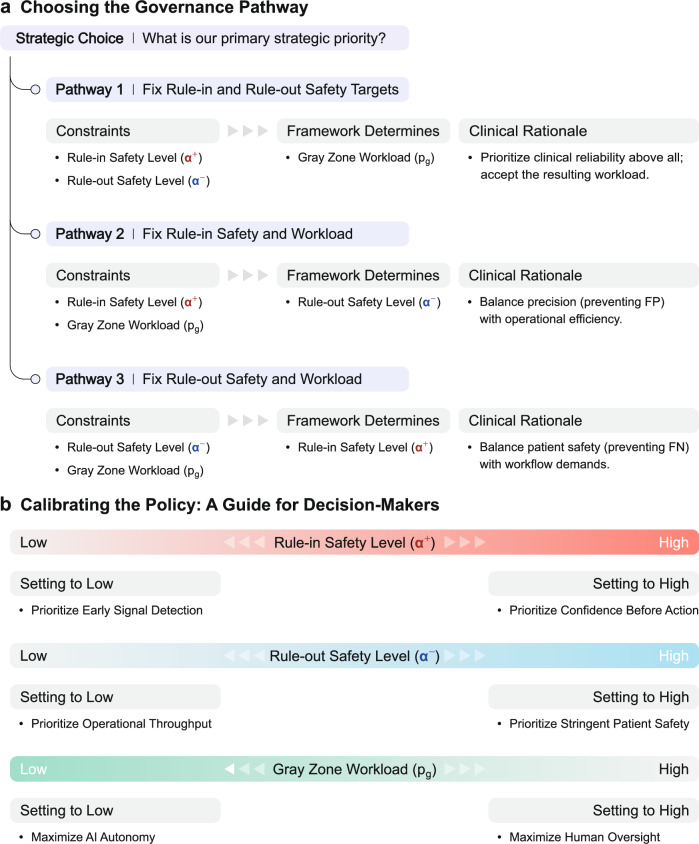
Framework for deriving operational policies. **a** Three governance pathways for policy formulation. Each policy is determined by constraining any two of the three parameters: rule-in safety level (*α*^+^), rule-out safety level (*α*⁻), or workload (i.e., the proportion of cases deferred to the Gray Zone, *p*_g_). **b** Conceptual illustration of the strategic trade-offs encountered when adjusting the parameters between ‘low’ and ‘high’ settings. FP: false positive, FN: false negative.

The first goal-driven paradigm, policy definition via direct constraints (Fig. 8), allows users to derive a policy from the interplay between three key parameters: rule-in reliability (governed by *α*^+^), rule-out reliability (governed by *α*⁻), and the clinical workload (represented by the proportion of the cohort deferred to the Gray Zone, *p*_g_). By defining any two of these as explicit constraints, the framework automatically optimizes for the third (Fig. 8a). For instance, a user might prioritize high safety standards for both ruling in and ruling out cases by setting targets for *α*^+^ and *α*⁻. The framework then calculates the resulting Gray Zone size, which represents the workload required to meet those dual-safety goals. Alternatively, a user could set a primary safety target (e.g., a high *α*⁻ for ruling out cases) and a maximum acceptable workload, allowing the framework to determine the best possible rule-in reliability (*α*^+^) achievable under those constraints. A conceptual guide to the strategic trade-offs involved in calibrating the ‘low’ versus ‘high’ settings for each parameter is provided in Fig. 8b. Examples of how different clinical scenarios (e.g., high-volume screening or pure cohort extraction for research, etc.) translate into specific policy constraints are provided in Supplementary Table 1.

**Fig. 9:**
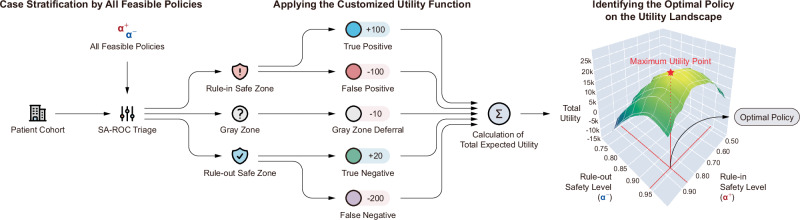
Conceptual diagram of the utility maximization process for optimal policy identification. The SA-ROC triage mechanism evaluates all feasible policies, each defined by a pair of safety levels (*α*^+^ and *α*⁻). For each policy under consideration, patients are stratified into the Rule-in Safe Zone, Gray Zone, or Rule-out Safe Zone. Comparing these zone assignments with the reference standard yields five potential outcomes for each patient: true positive, false positive, Gray Zone deferral, true negative, or false negative. The customized utility function, using the values pre-defined by the decision-maker, assigns specific scores to each of these outcomes. The calculation of total expected utility then sums these utility scores across all patients in the cohort for that specific policy. Finally, the framework searches across all feasible policies (the entire landscape of *α*^+^ and *α*⁻ combinations) to identify the optimal policy corresponding to the maximum utility point on the utility landscape.

The second utility-driven paradigm, policy derivation via utility maximization (Fig. 9), reframes policy design as an optimization problem that balances the benefits of correct decisions against the costs of errors and indecision. The process operates by assigning a specific clinical or economic value, or utility, to each of the five possible outcomes for a case: a true positive (TP, Utility *U*_TP_), true negative (TN, Utility *U*_TN_), false positive (FP, Utility *U*_FP_), false negative (FN, Utility *U*_FN_), and deferral to the Gray Zone (Utility, *U*_Gray_). Users can assign positive utility to correct decisions and negative utility (penalties) to errors, such as a high penalty for a missed cancer (a false negative). Examples of how these utility values can be assigned to reflect different clinical policy goals (e.g., Minimize FN vs. Minimize FP Impact, etc.) are provided in Supplementary Table 2. The framework then systematically identifies the policy that maximizes the total expected utility across all cases. For a policy defined by a pair of score thresholds, the total expected utility (*U*_Total_) is the weighted sum of the outcomes:

5 $${U}_{{\rm{Total}}}={n}_{\mathrm{TP}}{U}_{\mathrm{TP}}+{n}_{\mathrm{TN}}{U}_{\mathrm{TN}}+{n}_{\mathrm{FP}}{U}_{\mathrm{FP}}+{n}_{\mathrm{FN}}{U}_{\mathrm{FN}}+{n}_{\mathrm{Gray}}{U}_{\mathrm{Gray}}$$

where n is the number of cases in each respective category. The framework performs a systematic search across all valid threshold pairs to find the policy that maximizes this function.

### Simulation studies

We conducted simulation studies to demonstrate how the SA-ROC framework reveals operational differences obscured by traditional metrics (Fig. 2). We generated synthetic datasets to simulate the continuous risk scores (ranging from 0 to 1) produced by clinical AI models. Each dataset was created by drawing 5,000 samples for the negative class (*C*_0_) and 5,000 for the positive class (*C*_1_) from pairs of Beta distributions^51^. Specifically, we employed fixed distribution parameters selected to generate a wide variety of score distribution shapes. This approach allowed us to observe distinct operational behaviors of the Safe and Gray Zone, ranging from wide uncertain regions to narrow effective automation zones, under controlled theoretical conditions. From these generated models, we selected four representative scenarios to highlight key principles. These scenarios included models with modest overall performance but skewed distributions that produced either a strong Rule-in Safe Zone or a strong Rule-out Safe Zone. Additionally, we created two scenarios with nearly identical high AUCs of 0.960 but contrasting distribution overlaps, resulting in one model with a wide Gray Zone (indicating high uncertainty) and another with a narrow Gray Zone (indicating higher potential for safe automation). These curated scenarios were designed to clearly show how a model’s underlying score distribution, not just its aggregate AUC, dictates its practical operational safety profile.

### Retrospective validation using FDA-cleared AI solutions

To validate our framework’s utility on a real-world application, we performed a retrospective analysis of two anonymized, commercially available, FDA-cleared AI solutions for breast cancer detection. These were referred to as AI Solution #1 and AI Solution #2. While specific architectural details are proprietary, both solutions are FDA-cleared deep learning algorithms based on CNN architectures, trained on large clinical datasets to analyze mammography images. Both AI solutions take a mammogram as input and output a continuous risk score ranging from 0 to 1, representing the likelihood of breast cancer. Their inclusion serves to demonstrate the SA-ROC framework’s utility as a model-agnostic tool for evaluating diverse, clinically adopted AI solutions, operating irrespective of their internal implementation differences. This study was conducted under the approval of the Massachusetts General Hospital Institutional Review Board (IRB No. 2023P003130), with a waiver for patient informed consent. The dataset comprised 1000 screening mammograms with a 40% disease prevalence (400 malignant and 600 benign cases), where the reference standard for malignancy was established by biopsy and for benign cases by long-term radiological follow-up. In parallel, the initial diagnostic assessment by the original reading radiologist for each case was also collected to serve as a human performance benchmark, with outcomes (true positive, true negative, false positive, and false negative) determined against the same definitive reference standard. We applied the SA-ROC framework to the risk scores from both AI solutions, and to ensure statistical robustness, 95% confidence intervals for all key metrics, including AUC and the Γ_Area_ values derived at fixed safety levels (e.g., 90%, 95%, 100%), were estimated via a nonparametric percentile bootstrap procedure. Specifically, 2000 bootstrap samples were generated by resampling with replacement from the dataset. For each bootstrap sample, the metrics were recalculated, and the 2.5th and 97.5th percentiles of the empirical distribution of each metric were taken as the bounds of the 95% confidence interval. Furthermore, to analyze operational stability under varying prevalence conditions (1%–40%), we conducted a separate simulation analysis (see Supplementary Note 1). Based on the assumption of fixed class-conditional score distributions, we generated synthetic cohorts to empirically derive bootstrap-based confidence intervals. Specifically, to maximize the effective sample size of the reference distribution, we held the complete negative cohort constant (N = 600) as a stable baseline and generated synthetic cohorts by resampling positive cases with replacement to match the target disease prevalence.

## Supplementary information

Supplementary information

## Data Availability

The data that support the findings of this study are available from the corresponding author upon reasonable request. Access to the data is subject to institutional regulations and data use agreements due to the inclusion of clinical information. Requests will be evaluated to ensure compliance with privacy and ethical standards, and qualified researchers may obtain access for non-commercial academic use.

## Code availability

The code used to implement the SA-ROC framework is available at https://github.com/MGH-LMIC/SA-ROC. The code is written in Python 3.9.

## References

[1] Sharma, M. et al. Artificial intelligence applications in health care practice: scoping review. *J. Med. Internet Res.* **24**, e40238 (2022). 36197712 10.2196/40238 PMC9582911

[2] Marwaha, J. S. & Kvedar, J. C. Crossing the chasm from model performance to clinical impact: the need to improve implementation and evaluation of AI. *NPJ Digit. Med.* **5**, 25 (2022). 35241790 10.1038/s41746-022-00572-2 PMC8894388

[3] van de Sande, D. et al. To warrant clinical adoption AI models require a multi-faceted implementation evaluation. *NPJ Digit. Med.* **7**, 58 (2024). 38448743 10.1038/s41746-024-01064-1 PMC10918103

[4] Cabitza, F., Campagner, A. & Balsano, C. Bridging the “last mile” gap between AI implementation and operation: “data awareness” that matters. *Ann. Transl. Med.* **8**, 501 (2020). 10.21037/atm.2020.03.63 PMC7210125 32395545

[5] Seneviratne, M. G., Shah, N. H. & Chu, L. Bridging the implementation gap of machine learning in healthcare. *BMJ Innov* **6**, 45–47 (2020).

[6] Kompa, B., Snoek, J. & Beam, A. L. Second opinion needed: communicating uncertainty in medical machine learning. *NPJ Digit. Med.* **4**, 4 (2021). 33402680 10.1038/s41746-020-00367-3 PMC7785732

[7] Chua, M. et al. Tackling prediction uncertainty in machine learning for healthcare. *Nat. Biomed. Eng.* **7**, 711–718 (2023). 36581695 10.1038/s41551-022-00988-x

[8] McMahon, G. T. The risks and challenges of artificial intelligence in endocrinology. *J. Clin. Endocrinol. Metab.* **109**, e1468–e1471 (2024). 38471009 10.1210/clinem/dgae017

[9] Topol, E. J. High-performance medicine: the convergence of human and artificial intelligence. *Nat. Med.* **25**, 44–56 (2019). 30617339 10.1038/s41591-018-0300-7

[10] Vickers, A. J. & Elkin, E. B. Decision curve analysis: a novel method for evaluating prediction models. *Med. Decis. Making* **26**, 565–574 (2006). 17099194 10.1177/0272989X06295361 PMC2577036

[11] Swaminathan, A. et al. Selective prediction for extracting unstructured clinical data. *J. Am. Med. Inform. Assoc.* **31**, 188–197 (2024). 10.1093/jamia/ocad182 PMC10746316 37769323

[12] Luo, R. et al. Sample-efficient safety assurances using conformal prediction. *Int. J. Robot. Res.* **43**, 1409–1424 (2024).

[13] Carlevaro, A., Alamo, T., Dabbene, F. & Mongelli, M. Probabilistic safety regions via finite families of adjustable classifiers. *IEEE Trans. Neural Netw. Learn. Syst.* **36**, 16198–16212 (2025). 10.1109/TNNLS.2025.3568174 40408201

[14] Narteni, S., Carlevaro, A., Dabbene, F., Muselli, M. & Mongelli, M. A novel score function for conformal prediction in rule-based binary classification. *Pattern Recognit*. 112219 (2025).

[15] Sreenivasan, A. P. et al. Conformal prediction enables disease course prediction and allows individualized diagnostic uncertainty in multiple sclerosis. *NPJ Digit. Med.* **8**, 224 (2025). 40275055 10.1038/s41746-025-01616-z PMC12022056

[16] Dalal, S., Ardabili, A. K. & Bonavia, A. S. Time-series deep learning and conformal prediction for improved sepsis diagnosis in primarily non-ICU hospitalized patients. *Comput. Biol. Med.* **193**, 110497 (2025). 10.1016/j.compbiomed.2025.110497 PMC12174992 40450820

[17] Fayyad, J., Alijani, S. & Najjaran, H. Empirical validation of conformal prediction for trustworthy skin lesions classification. *Comput. Methods Programs Biomed.* **253**, 108231 (2024). 38820714 10.1016/j.cmpb.2024.108231

[18] Sadinle, M., Lei, J. & Wasserman, L. Least ambiguous set-valued classifiers with bounded error levels. *J. Am. Stat. Assoc.* **114**, 223–234 (2019).

[19] Deng, J. et al. So you’ve got a high AUC, now what? An overview of important considerations when bringing machine-learning models from computer to bedside. *Med. Decis. Making* **45**, 640–653 (2025). 10.1177/0272989X251343082 PMC12260203 40439482

[20] Kelly, C. J., Karthikesalingam, A., Suleyman, M., Corrado, G. & King, D. Key challenges for delivering clinical impact with artificial intelligence. *BMC Med* **17**, 195 (2019). 31665002 10.1186/s12916-019-1426-2 PMC6821018

[21] Alam, L. & Mueller, S. Examining the effect of explanation on satisfaction and trust in AI diagnostic systems. *BMC Med. Inform. Decis. Mak.* **21**, 178 (2021). 34082719 10.1186/s12911-021-01542-6 PMC8176739

[22] Afroogh, S., Akbari, A., Malone, E., Kargar, M. & Alambeigi, H. Trust in AI: progress, challenges, and future directions. *Humanit. Soc. Sci. Commun.* **11**, 1568 (2024).

[23] Hassan, M., Kushniruk, A. & Borycki, E. Barriers to and facilitators of artificial intelligence adoption in health care: scoping review. *JMIR Hum. Factors* **11**, e48633 (2024). 39207831 10.2196/48633 PMC11393514

[24] Hanley, J. A. & McNeil, B. J. The meaning and use of the area under a receiver operating characteristic (ROC) curve. *Radiology* **143**, 29–36 (1982). 7063747 10.1148/radiology.143.1.7063747

[25] Swamidass, S. J., Azencott, C.-A., Daily, K. & Baldi, P. A CROC stronger than ROC: measuring, visualizing and optimizing early retrieval. *Bioinformatics* **26**, 1348–1356 (2010). 20378557 10.1093/bioinformatics/btq140 PMC2865862

[26] Chakraborty, D. P. & Winter, L. Free-response methodology: alternate analysis and a new observer-performance experiment. *Radiology* **174**, 873–881 (1990). 2305073 10.1148/radiology.174.3.2305073

[27] Begoli, E., Bhattacharya, T. & Kusnezov, D. The need for uncertainty quantification in machine-assisted medical decision making. *Nat. Mach. Intell.* **1**, 20–23 (2019).

[28] Do, S. Explainable & safe artificial intelligence in radiology. *J. Korean Soc. Radiol.* **85**, 834–847 (2024). 10.3348/jksr.2024.0118 PMC11473981 39416324

[29] Kim, D. et al. Accurate auto-labeling of chest X-ray images based on quantitative similarity to an explainable AI model. *Nat. Commun.* **13**, 1867 (2022). 35388010 10.1038/s41467-022-29437-8 PMC8986787

[30] Yoon, B. C. et al. Incorporating algorithmic uncertainty into a clinical machine deep learning algorithm for urgent head CTs. *PLoS One* **18**, e0281900 (2023). 36913348 10.1371/journal.pone.0281900 PMC10010506

[31] Geifman, Y. & El-Yaniv, R. SelectiveNet: A deep neural network with an integrated reject option. *Proc. Mach. Learn. Res.* **97**, 2151–2159 (2019).

[32] Shafer, G. & Vovk, V. A tutorial on conformal prediction. *J. Mach. Learn. Res.* **9**, 371–421 (2008).

[33] Angelopoulos, A. N. & Bates, S. Conformal prediction: a gentle introduction. *Found. Trends Mach. Learn.* **16**, 494–591 (2023).

[34] Angelopoulos, A. N., Bates, S., Fisch, A., Lei, L. & Schuster, T. Conformal risk control. *Proc. Int. Conf. Learn. Represent.* 55198–55218, (2024).

[35] Liu, H. et al. Artificial intelligence and radiologist burnout. *JAMA Netw. Open* **7**, e2448714 (2024). 39576636 10.1001/jamanetworkopen.2024.48714 PMC11584928

[36] Tai-Seale, M. et al. AI-generated draft replies integrated into health records and physicians’ electronic communication. *JAMA Netw. Open* **7**, e246565 (2024). 38619840 10.1001/jamanetworkopen.2024.6565 PMC11019394

[37] Ong Ly, C. et al. Shortcut learning in medical AI hinders generalization: method for estimating AI model generalization without external data. *NPJ Digit. Med.* **7**, 124 (2024). 38744921 10.1038/s41746-024-01118-4 PMC11094145

[38] Eche, T., Schwartz, L. H., Mokrane, F.-Z. & Dercle, L. Toward generalizability in the deployment of artificial intelligence in radiology: role of computation stress testing to overcome underspecification. *Radiol. Artif. Intell.* **3**, e210097 (2021). 34870222 10.1148/ryai.2021210097 PMC8637230

[39] Ciecierski-Holmes, T., Singh, R., Axt, M., Brenner, S. & Barteit, S. Artificial intelligence for strengthening healthcare systems in low- and middle-income countries: a systematic scoping review. *NPJ Digit. Med.* **5**, 162 (2022). 10.1038/s41746-022-00700-y PMC9614192 36307479

[40] Alami, H. et al. Artificial intelligence in health care: laying the foundation for responsible, sustainable, and inclusive innovation in low- and middle-income countries. *Global. Health* **16**, 52 (2020). 32580741 10.1186/s12992-020-00584-1 PMC7315549

[41] Oduoye, M. O. et al. Impacts of the advancement in artificial intelligence on laboratory medicine in low- and middle-income countries: challenges and recommendations—a literature review. *Health Sci. Rep.* **7**, e1794 (2024). 10.1002/hsr2.1794 PMC10766873 38186931

[42] Fernandes, C. O., Miles, S., Lucena, C. J. P. D. & Cowan, D. Artificial intelligence technologies for coping with alarm fatigue in hospital environments because of sensory overload: algorithm development and validation. *J. Med. Internet Res.* **21**, e15406 (2019). 31769762 10.2196/15406 PMC6904899

[43] Grignoli, N. et al. Clinical decision fatigue: a systematic and scoping review with meta-synthesis. *Fam. Med. Community Health* **13**, e003033 (2025). 39922690 10.1136/fmch-2024-003033 PMC11808891

[44] Abdelwanis, M., Alarafati, H. K., Tammam, M. M. S. & Simsekler, M. C. E. Exploring the risks of automation bias in healthcare artificial intelligence applications: A Bowtie analysis. *J. Saf. Sci. Resil.* **5**, 460–469 (2024).

[45] Cestonaro, C., Delicati, A., Marcante, B., Caenazzo, L. & Tozzo, P. Defining medical liability when artificial intelligence is applied on diagnostic algorithms: a systematic review. *Front. Med.* **10**, 1305756 (2023). 10.3389/fmed.2023.1305756 PMC10711067 38089864

[46] Buçinca, Z., Malaya, M. B. & Gajos, K. Z. To trust or to think: cognitive forcing functions can reduce overreliance on AI in AI-assisted decision-making. *Proc. ACM Hum. Comput. Interact.* **5**, 1–21 (2021).

[47] Han, P. K. et al. How physicians manage medical uncertainty: a qualitative study and conceptual taxonomy. *Med. Decis. Making* **41**, 275–291 (2021). 33588616 10.1177/0272989X21992340 PMC7985858

[48] Mohammadi, F. G. & Sebro, R. Artificial intelligence impact on burnout in radiologists—alleviation or exacerbation?. *JAMA Netw. Open* **7**, e2448720 (2024). 10.1001/jamanetworkopen.2024.48720 39576648

[49] Feng, J. et al. Clinical artificial intelligence quality improvement: towards continual monitoring and updating of AI algorithms in healthcare. *NPJ Digit. Med.* **5**, 66 (2022). 35641814 10.1038/s41746-022-00611-y PMC9156743

[50] Bruno, P., Quarta, A. & Calimeri, F. Continual learning in medicine: a systematic literature review. *Neural Process. Lett.* **57**, 2 (2025).

[51] Gupta, A. K. & Nadarajah, S. *Handbook of Beta Distribution and Its Applications* (CRC press, Boca Raton, FL USA, 2004).

