# Supplementary material for: Defining operational safety in clinical artificial intelligence systems
Source: NPJ Digit Med. 2026 Feb 20;9:281. doi: 10.1038/s41746-026-02450-7 (PMC13050326; doi:10.1038/s41746-026-02450-7)
Supplement: Supplementary file 1 — Supplementary information [file 41746_2026_2450_MOESM1_ESM.pdf]

Supplementary Note 1:
Analysis of Operational Stability Under Varying Disease Prevalence

Simulation Design

The safety zones defined by the SA-ROC framework are based on PPV and NPV—metrics known to be prevalence-dependent. Consequently, the operational stability of these zones can be influenced by shifts in prevalence. To assess the generalizability of our findings and the models' operational stability under these shifts, we conducted a simulation analysis by systematically varying disease prevalence.

To rigorously test this, we started with our full dataset of 1,000 cases. We adopted a controlled resampling strategy designed to maximize statistical robustness given the finite dataset size. Specifically, we generated synthetic datasets to explicitly capture the sampling variability inherent in the positive class.

Crucially, we established the complete set of 600 negative cases as a fixed reference distribution. This design choice was made to preserve the full resolution of the majority class, avoiding the information loss or added variance that would result from downsampling or resampling the negative cohort. The simulation proceeded as follows:

- Baseline Preservation: The 600 negative cases were held constant across all iterations to serve as a stable anchor.
- Targeted Resampling Calculation: To simulate prevalence varying from 1% to 40% (in 1% increments), we first calculated the corresponding number of positive cases required relative to the fixed negative baseline. For example, a 10% prevalence required ~67 positive cases to be added to the 600 negative cases.
- Bootstrap Generation: We generated 2,000 bootstrap resamples for each prevalence step. Each resample was created by drawing the required number of positive cases with replacement from the original pool of 400 positive cases, and then combining them with the fixed set of 600 negative cases.

This procedure allowed for robust estimation of the safety zones and their 95% confidence intervals at each prevalence level, isolating the mechanical effect of prevalence shifts while utilizing the maximum available sample size.

Trends in Operational Stability across Prevalence Shifts

This simulation analysis reveals that a model's operational stability is fundamentally dictated by its intrinsic score distribution morphology. FDA-cleared AI Solution #1 exhibited a pattern of conditional stability. At the most stringent safety level (α = 100%), its operational safety was notably sensitive to prevalence shifts. The Gray Zone Area (Γ_{Area}) expanded rapidly as prevalence increased from 1% to 15%, eventually stabilizing at a high level of uncertainty (Supplementary Figure 1). This instability arises because FDA-cleared AI Solution #1's score distribution for positive cases includes outliers with very low predicted risk scores. As prevalence increases, these "hard-to-classify" positive cases are more frequently included in the bootstrapped samples. To satisfy the α = 100% policy, the Rule-out Safe Zone is forced to contract progressively, which in turn reclassifies a large volume of true negative cases into the Gray Zone, causing the Γ_{Area} to spike. However, when the safety requirements were relaxed to α = 95% and α = 90%, the system became remarkably stable and operationally efficient, demonstrating a consistently low Γ_{Area}.

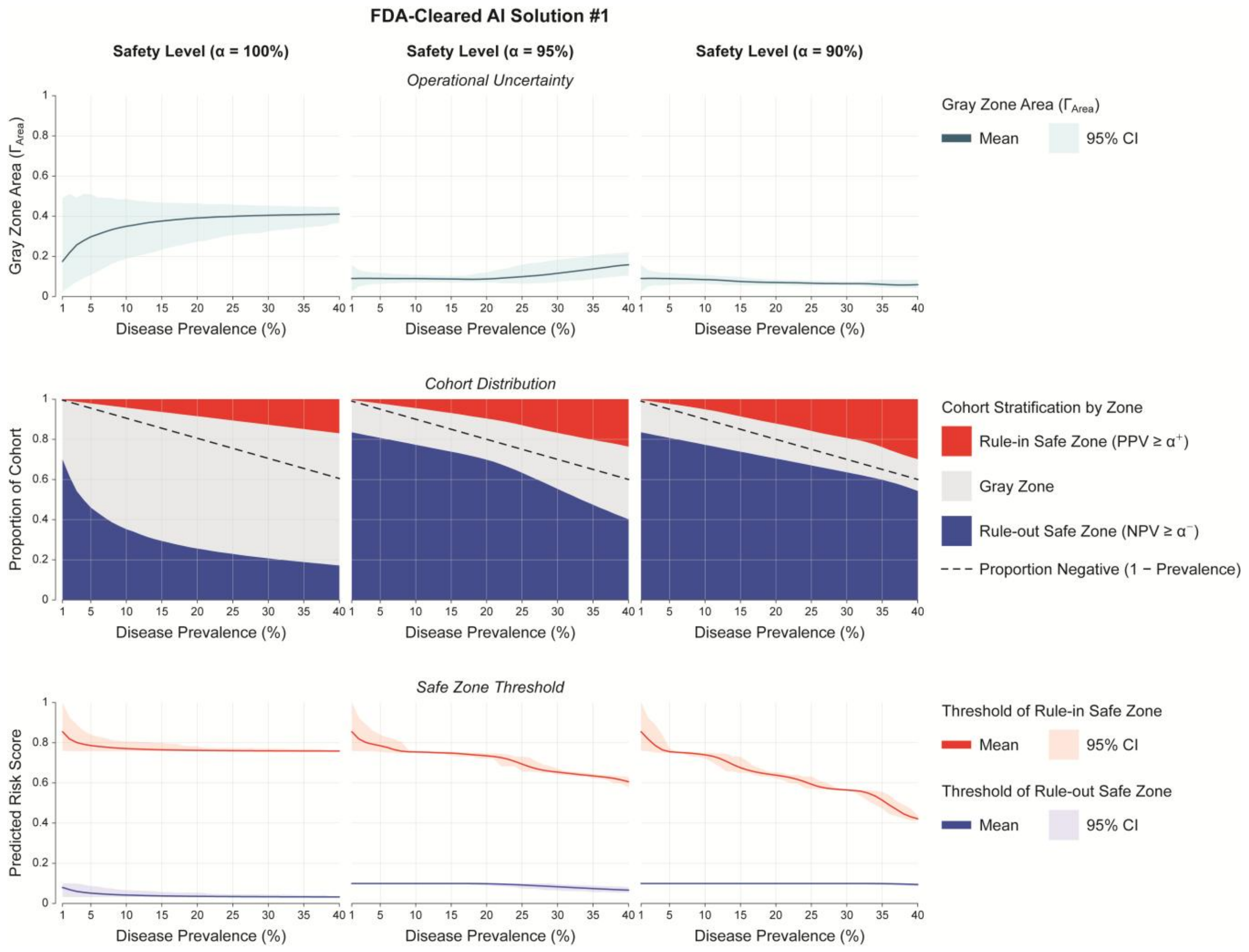

Supplementary Figure 1: Analysis of operational safety across varying disease prevalence for FDA-cleared AI Solution #1. (Top Row) Gray Zone Area (Γ_{Area}) as a function of prevalence at three safety levels (α = 100%, 95%, 90%). (Middle Row) Proportion of the cohort stratified into Rule-in Safe, Rule-out Safe, and Gray Zones; the dashed line serves as a reference indicating the proportion of negative cases (1 – prevalence). (Bottom Row) Predicted risk score thresholds defining the boundaries of the safe zones. Shaded areas represent 95% confidence intervals (CI).

In contrast, FDA-cleared AI Solution #2 demonstrated a high degree of robustness. While its Γ_{Area} was generally high at moderate safety levels (α = 95%, 90%), its defining characteristic was its high stability (Supplementary Figure 2). Its operational performance and Γ_{Area} remained highly stable and consistent across all tested safety levels (α = 100%, 95%, 90%), showing almost no variation regardless of disease prevalence. This stability is a direct result of its well-behaved score distribution, which lacks the extreme low-scoring positive outliers. Because there are no such challenging cases, the Rule-out Safe Zone boundary does not need to shift drastically to maintain high NPV, even at the α = 100% level.

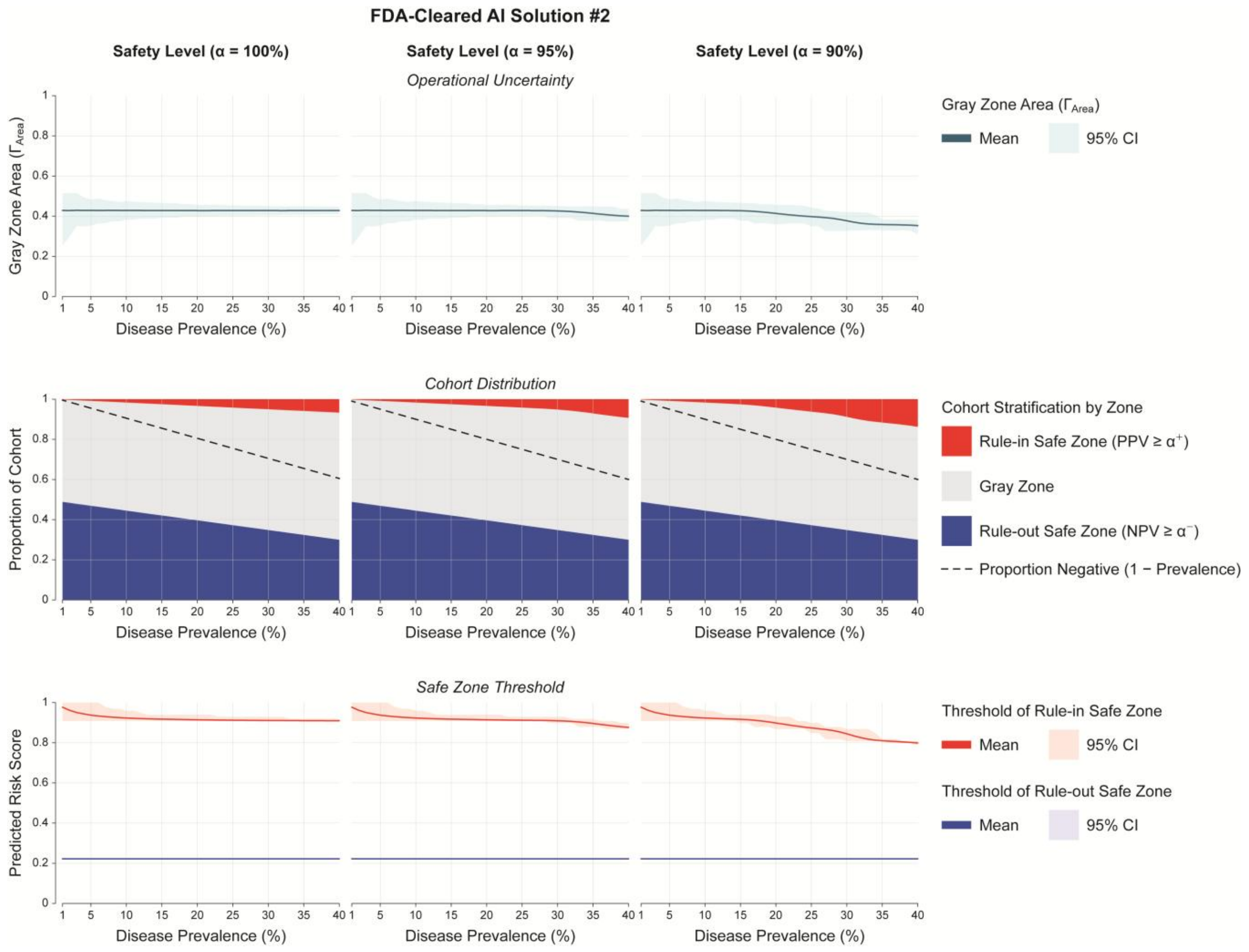

Supplementary Figure 2: Analysis of operational safety across varying disease prevalence for FDA-cleared AI Solution #2. (Top Row) Gray Zone Area (Γ_{Area}) as a function of prevalence at three safety levels (α = 100%, 95%, 90%). (Middle Row) Proportion of the cohort stratified into Rule-in Safe, Rule-out Safe, and Gray Zones; the dashed line serves as a reference indicating the proportion of negative cases (1 – prevalence). (Bottom Row) Predicted risk score thresholds defining the boundaries of the safe zones. Shaded areas represent 95% confidence intervals (CI).

This simulation analysis reveals a key insight: while the sensitivity of PPV and NPV to prevalence is a known statistical principle, the degree to which this translates into operational instability is critically governed by the morphology of the model's score distribution. The SA-ROC framework allows for the dissociation of these effects, revealing the complex interplay between epidemiological context (prevalence) and a model's intrinsic distributional characteristics.

Dynamics of the Rule-out Safe Zone and Performance Reversal

To directly compare the operational utility under varying epidemiological conditions, we analyzed the capacity of the Rule-out Safe Zone (α^{-} = 100%) for both models (Supplementary Figure 3). This analysis revealed a distinct crossover at approximately 2.48% prevalence. Below this threshold, AI #1 showed a marginal advantage. However, beyond 2.48%, the trend reversed, with AI #2 consistently maintaining a larger Rule-out Safe Zone than AI #1. Importantly, this gap in operational capacity widened as prevalence increased, achieving statistical significance ($P < 0.05$, two-sided non-parametric paired bootstrap test) at prevalences of 21% and above. This confirms that the ability of AI #2 to safely reduce workload is a robust characteristic in moderate-to-high prevalence settings.

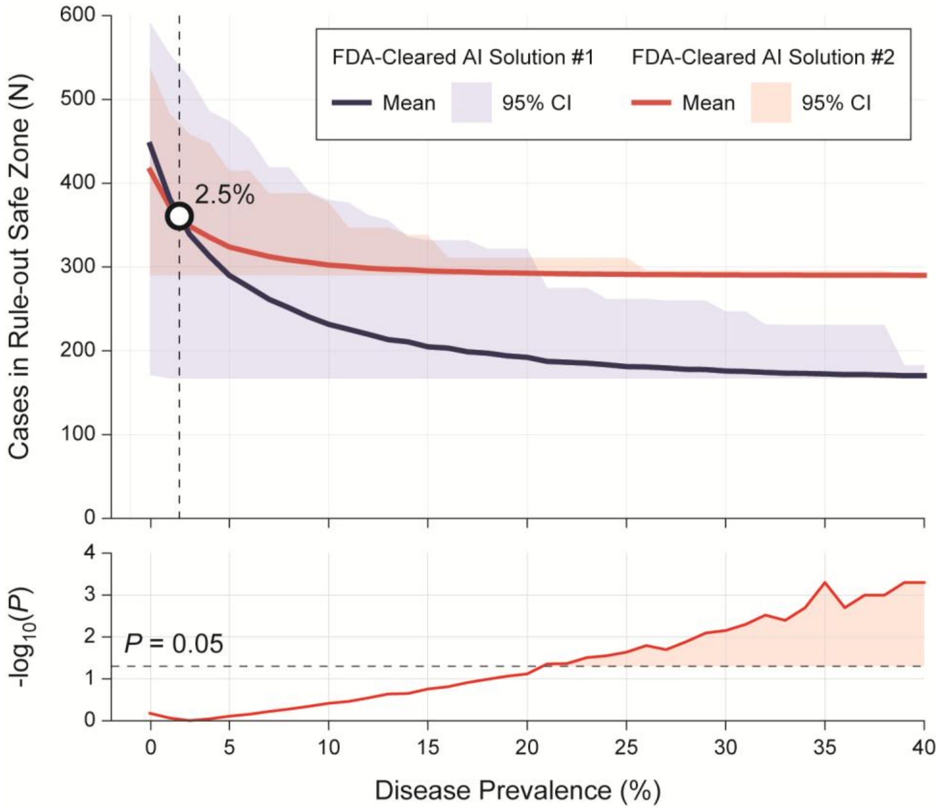

Supplementary Figure 3: Comparative operational stability of the Rule-out Safe Zone under varying disease prevalence. The figure displays the mean number of cases correctly ruled out at the maximal safety level (α^{-} = 100%) and the statistical significance of the difference between the two models, expressed as $-\log_{10}(P)$, across disease prevalence scenarios ranging from 1% to 40%. The simulation employed a fixed negative cohort (N = 600) with resampled positive cases (2,000 bootstrap iterations). The open circle marks the performance crossover point at approximately 2.5% prevalence. The horizontal dashed line represents the threshold for statistical significance ($P = 0.05$; two-sided non-parametric paired bootstrap test). Shaded areas indicate 95% confidence intervals.

Supplementary Table 1: Example scenarios for setting clinical policy constraints (α^+, α^- and p_g).

Scenario	Policy Objective	Policy Constraints (Inputs)	Rationale for Constraint Selection
Moderate Bidirectional Confidence	To achieve high rule-out confidence for de-prioritization, while a moderate rule-in is sufficient for guiding evaluation.	• $\alpha^+ = 85\%$ (Moderate Rule-in PPV) • $\alpha^- = 95\%$ (High Rule-out NPV)	(Corresponds to Fig. 5a) This policy reflects a balanced clinical need. The constraints prioritize a high-reliability rule-out (NPV ≥ 95%) to safely de-prioritize a large volume of cases, while also accepting a moderate rule-in (PPV ≥ 85%) as sufficient for flagging cases for review. The resulting workload (p_g) is the cost of meeting both safety targets.
High Rule-in Precision	To minimize diagnostic interruptions and unnecessary workload by flagging only cases with near-certain malignancy.	• $\alpha^+ = 99\%$ (Near-perfect Rule-in PPV) • $p_g = 20\%$ (Max Workload)	(Corresponds to Fig. 5b) This policy is for high-stakes diagnoses where a false positive (FP) is highly disruptive. The primary constraint is a near-perfect PPV ($\alpha^+ = 99\%$). A maximum acceptable workload ($p_g \leq 20\%$) is also set to ensure operational efficiency. The framework then derives the best possible α^- (Rule-out NPV) achievable under these two constraints.
Maximizing Rule-out Confidence	To ensure all 'negative' results are truly reliable, mandating human review for any ambiguous case to guarantee safety.	• $\alpha^- = 99\%$ (Near-perfect Rule-out NPV) • $p_g = 50\%$ (Max Workload)	(Corresponds to Fig. 5c) A classic screening policy where minimizing false negatives (FN) is the absolute priority. It sets a near-perfect NPV ($\alpha^- = 99\%$) as the primary constraint. A high but fixed ceiling for workload ($p_g \leq 50\%$) is deemed acceptable to achieve this safety goal. The resulting rule-in reliability (α^+) is then determined by the framework.
High-Volume Screening	To maximize workflow efficiency by safely ruling out the vast majority of negative cases while minimizing the human review workload.	• $\alpha^- = 99\%$ (Near-perfect Rule-out NPV) • $p_g = 5\%$ (Very Low Max Workload)	This is an efficiency-focused screening policy. It mandates both high safety (NPV ≥99%) and extremely high automation by strictly limiting the Gray Zone workload to 5% of the cohort. This policy accepts that the resulting rule-in precision (α^+) may be lower as a trade-off for achieving this high level of workflow efficiency.
Pure Cohort Extraction for Research	To identify and extract "pure" positive and "pure" negative cohorts for research, prioritizing sample certainty over automation efficiency.	• $\alpha^+ = 100\%$ (Perfect Rule-in PPV) • $\alpha^- = 100\%$ (Perfect Rule-out NPV)	This policy is not for clinical automation but for research sample selection. The goal is to extract cohorts with the highest possible confidence. Both α^+ and α^- are set to perfect levels. The resulting workload (p_g) is expected to be very large, as any case with even slight ambiguity is, by design, filtered into the Gray Zone and excluded from the study.

α^+ (Rule-in safety level): The minimum acceptable positive predictive value (PPV) required to trust a positive prediction; α^- (Rule-out safety level): The minimum acceptable negative predictive value (NPV) required to trust a negative prediction; p_g (Gray Zone percentage): The percentage of the total cohort that falls into the Gray Zone, representing the human review workload. A policy is defined by setting constraints for any two of these three parameters (α^+, α^-, p_g), and the framework then optimizes for the third.

Supplementary Table 2: Example utility assignments reflecting different clinical policy goals.

Scenario	Outcome	Assigned Utility	Rationale for Utility Assignments
Balanced Utility (Careful Decision Policy)	TP	+800	(Corresponds to Fig. 5d) A correct diagnosis holds significant clinical value, outweighing moderate costs of errors or review. The policy aims for a sensible balance between detecting disease and avoiding unnecessary actions, reflecting standard diagnostic practice where both sensitivity and specificity are important.
	TN	+50	
	FP	-500	
	FN	-500	
	C_g	-5	
Strictly Non-Negligent (Minimize FN)	TP	+10	(Corresponds to Fig. 5e) The absolute priority is to avoid missing any cancer case (FN). The assigned utilities reflect this extreme risk aversion: the cost of a false negative (-1000) vastly outweighs any potential benefit or cost of other outcomes. Correct diagnoses (TP/TN) have minimal positive value compared to the overwhelming negative value of an FN. The cost of FP or Gray Zone deferral becomes negligible in the face of minimizing FN at all costs.
	TN	+10	
	FP	-10	
	FN	-1,000	
	C_g	0	
Minimize FP Impact (Prevent Patient Harm from FP)	TP	+100	(Corresponds to Fig. 5f) The primary goal is to prevent patient harm from false alarms (FP), recommending further action only when evidence is very clear. Thus, the cost of a false positive (-1000) is extremely high, significantly outweighing the cost of a missed case (-100) in this specific policy context. The value of correct decisions (TP/TN) is positive but secondary to avoiding FPs.
	TN	+20	
	FP	-1,000	
	FN	-100	
	C_g	0	
High-Volume Screening (Maximize Efficient Rule-out)	TP	+80	In mass screening, correctly ruling out the vast majority of negative cases automatically (high TN value) is crucial for efficiency. Missing a case (FN) remains highly undesirable (-1000), while false positives (-20) lead to recalls which are less costly than missed cancers. A small penalty was assigned to the Gray Zone (-10) to encourage automation.
	TN	+100	
	FP	-20	
	FN	-1,000	
	C_g	-10	
Resource-Limited Setting (Minimize Costly Errors)	TP	+50	Both false positives (-500, unnecessary resource use) and false negatives (-500, missed treatment opportunity) are highly costly. Correct decisions (TP/TN) provide value, but minimizing costly errors is paramount. Gray Zone deferral (-50) represents significant burden due to limited expert availability.
	TN	+50	
	FP	-500	
	FN	-500	
	C_g	-50	

TP: true positive; TN: true negative; FP: false positive; FN: false negative; C_g: cases deferred to the Gray Zone for additional review. All values are dimensionless, illustrative utilities that encode decision-maker preferences rather than being estimated from data. The optimal policy is invariant to positive affine transformations of utilities; only relative magnitudes matter.
